# Effect of spironolactone on cardiovascular and renal outcomes in patients with chronic kidney disease

**DOI:** 10.1093/ckj/sfaf247

**Published:** 2025-08-06

**Authors:** Tz-Heng Chen, Shuo-Ming Ou, Kuan-Hsun Lin, Yang Ho, Wei-Cheng Tseng, Yuan-Chia Chu, Der-Cherng Tarng

**Affiliations:** Division of Nephrology, Department of Medicine, Taipei Veterans General Hospital, Taipei, Taiwan; School of Medicine, National Yang Ming Chiao Tung University, Taipei, Taiwan; Institute of Emergency and Critical Care Medicine, National Yang Ming Chiao Tung University, Taipei, Taiwan; Division of Nephrology, Department of Medicine, Taipei Veterans General Hospital, Taipei, Taiwan; School of Medicine, National Yang Ming Chiao Tung University, Taipei, Taiwan; Institute of Clinical Medicine, National Yang Ming Chiao Tung University, Taipei, Taiwan; Department of Information Management, Taipei Veterans General Hospital, Taipei, Taiwan; Department of Information Management, National Taipei University of Nursing and, Health Sciences, Taipei, Taiwan; Division of Nephrology, Department of Medicine, Taipei Veterans General Hospital, Taipei, Taiwan; School of Medicine, National Yang Ming Chiao Tung University, Taipei, Taiwan; Division of Nephrology, Department of Medicine, Taipei Veterans General Hospital, Taipei, Taiwan; School of Medicine, National Yang Ming Chiao Tung University, Taipei, Taiwan; Department of Information Management, Taipei Veterans General Hospital, Taipei, Taiwan; Department of Information Management, National Taipei University of Nursing and, Health Sciences, Taipei, Taiwan; Big Data Center, Taipei Veterans General Hospital, Taipei, Taiwan; Division of Nephrology, Department of Medicine, Taipei Veterans General Hospital, Taipei, Taiwan; School of Medicine, National Yang Ming Chiao Tung University, Taipei, Taiwan; Department and Institute of Physiology, National Yang Ming Chiao Tung University, Taipei, Taiwan

**Keywords:** cardiovascular outcomes, chronic kidney disease, end-stage renal disease, mortality, spironolactone

## Abstract

**Background:**

Steroidal mineralocorticoid receptor antagonists (MRAs), including spironolactone, effectively treat resistant hypertension, reduce proteinuria and lower mortality in heart failure with reduced ejection fraction. However, their long-term effects in chronic kidney disease (CKD) remain unclear. This study investigated spironolactone's impact on end-stage renal disease (ESRD), major adverse cardiovascular events (MACE), hyperkalemia and mortality in CKD patients.

**Methods:**

This retrospective hospital-based cohort study enrolled patients with CKD stage 3–5 between 1 January 2011 and 30 June 2023. The patients were classified as spironolactone users or nonusers, with each user matched to two nonusers by propensity scores. The outcomes of interest included ESRD, MACE, all-cause mortality and severe hyperkalemia. MACE include nonfatal stroke, nonfatal myocardial infarction and cardiovascular death.

**Results:**

After propensity score matching, 2711 spironolactone users and 5422 nonusers were included in this analysis. Spironolactone users exhibited higher risks of all-cause mortality [adjusted hazard ratio (aHR) 1.23; 95% confidence interval (CI) 1.11–1.37] and severe hyperkalemia (aHR 1.44; 95% CI 1.24–1.68) than nonusers. However, there was a lower risk of MACE (aHR 0.90; 95% CI 0.82–0.99), primarily due to a significant reduction in stroke risk (aHR 0.79; 95% CI 0.71–0.88). The risk of ESRD was similar between the two groups (aHR 1.09; 95% CI 0.85–1.38).

**Conclusions:**

In patients with CKD, spironolactone use was associated with a decreased risk of stroke but increased risks of severe hyperkalemia and all-cause mortality, while the risk of ESRD remained unchanged. Individualized clinical decision-making and appropriate dose adjustment are important to balance the potential benefits and risks associated with spironolactone therapy.

## GRAPHICAL ABSTRACT

**GRAPHICAL ABSTRACT:**
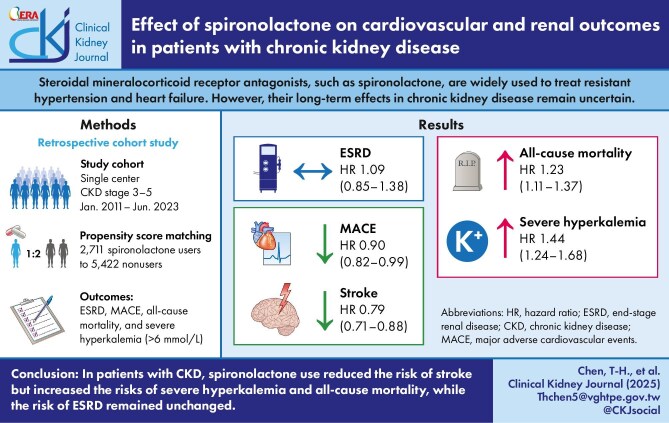

KEY LEARNING POINTS **What was known:**

- Overactivation of the mineralocorticoid receptor contributes to sodium retention, inflammation and fibrosis, accelerating chronic kidney disease (CKD) progression and cardiovascular disease.
- Spironolactone reduces proteinuria, treats resistant hypertension and improves outcomes in heart failure patients. However, its use in CKD has been limited due to concerns about hyperkalemia.
- Finerenone, a non-steroidal mineralocorticoid receptor antagonist, has shown cardiovascular and renal benefits in CKD patients with type 2 diabetes, but data on spironolactone's real-world use in broader CKD populations is limited.

**This study adds:**

- Spironolactone use in patients with CKD was associated with fewer cardiovascular events, particularly a reduced risk of stroke. However, it was also linked to increased all-cause mortality and a higher risk of severe hyperkalemia.
- No significant difference in the progression to end-stage renal disease was observed between spironolactone users and nonusers.

**Potential impact:**

- Patient selection and close monitoring are essential when using spironolactone in CKD to avoid hyperkalemia.
- Clinicians should carefully assess individual CKD patient risks when prescribing spironolactone, balancing its cerebroprotective benefits, particularly stroke prevention, against the elevated mortality risk.

## INTRODUCTION

Chronic kidney disease (CKD) has long been a significant global health issue, affecting approximately 10% of the global population, leading to significant morbidity and mortality [1–3]. Without effective treatment or prevention strategies, CKD progression is strongly associated with an increased risk of cardiovascular diseases, which is the leading cause of death in this patient population [4, 5]. Overactivation of the mineralocorticoid receptor is one of the key mechanisms in CKD progression, facilitating sodium retention, inflammation and fibrosis, which collectively exacerbate renal and cardiovascular diseases [6–8].

Conventional steroidal mineralocorticoid receptor antagonists (MRAs), including spironolactone, reduce mortality in patients with heart failure with reduced ejection fraction while also effectively treating resistant hypertension and lowering proteinuria [9–13]. However, the use of steroidal MRAs in patients with CKD is limited due to concerns regarding hyperkalemia, especially in those with advanced kidney disease [14, 15]. The recent introduction of finerenone, a new non-steroidal MRA, has drawn increased attention to the role of MRAs in CKD management. Finerenone has demonstrated efficacy in reducing cardiovascular and renal events in patients with CKD and type 2 diabetes, presenting a lower risk of hyperkalemia than steroidal MRAs such as spironolactone [16–18]. The therapeutic effects are primarily attributed to its antifibrotic and anti-inflammatory properties, which may slow CKD progression and reduce cardiovascular risks [19, 20]. Although finerenone represents a significant advancement, the long-term effects of spironolactone, particularly in an extensive population of patients with CKD without considering their diabetic status, remain underexplored.

We conducted a retrospective cohort study using real-world data to investigate the effect of spironolactone on the risks of developing end-stage renal disease (ESRD), major adverse cardiovascular events (MACE) and mortality among patients with CKD. Furthermore, we examined the risk of severe hyperkalemia.

## MATERIALS AND METHODS

### Methods

This retrospective hospital-based cohort study was conducted in Taipei Veterans General Hospital, a tertiary medical center. We enrolled patients with CKD stages 3–5 aged ≥20 years between 1 January 2011 and 30 June 2023. CKD stage was diagnosed based on estimated glomerular filtration rate (eGFR) values, according to the Kidney Disease: Improving Global Outcomes clinical practice guideline [21]. The eGFR was calculated using the 2021 Chronic Kidney Disease Epidemiology Collaboration equation [22]. Patients who were already receiving chronic renal replacement therapy, those who had undergone kidney transplantation or those receiving other types of MRA were excluded from the analysis. Baseline demographic data were obtained from the electronic medical records of the patients.

The patients were divided into spironolactone user and nonuser groups. Spironolactone users were those who had been receiving spironolactone for at least 30 consecutive days during the study, while the other patients were considered nonusers. To assess potential dose–response relationships, spironolactone exposure was further quantified by calculating both the cumulative dose and the daily mean dose throughout the follow-up period. Patients were followed until death or the end of the study, whichever occurred first. This study was approved by the Institutional Review Board of Taipei Veterans General Hospital (VGH-2023-07-001CC).

### Outcomes

The primary outcomes of interest included ESRD, MACE, all-cause mortality and severe hyperkalemia. ESRD was defined as the initiation of maintenance dialysis or kidney transplantation. MACE were defined as a composite of nonfatal stroke, nonfatal myocardial infarction and cardiovascular death. Severe hyperkalemia was defined as a serum potassium level >6.0 mmol/L.

### Management of severe hyperkalemia

For patients who developed severe hyperkalemia, the management strategies and their associations with recurrent hyperkalemia and adverse clinical outcomes were evaluated. Management strategies were defined as any intervention initiated within 90 days of the initial hyperkalemia episode, including potassium binder use, spironolactone dose reduction and spironolactone discontinuation. Recurrent hyperkalemia was defined as a serum potassium level >6.0 mmol/L occurring more than 90 days after the initial episode. Patients were classified into five groups according to the treatment received: no management, potassium binder only, spironolactone dose reduction only, spironolactone discontinuation only and combined management (both potassium binder and spironolactone adjustment).

### Statistical analysis

Continuous data were presented as means ± standard deviations for normally distributed variables and as medians with interquartile ranges for non-normally distributed variables. Categorical variables were presented as frequencies and percentages. Baseline characteristics of spironolactone users and nonusers were compared utilizing Pearson's chi-squared test for categorical variables, the independent t-test for parametric continuous variables, and the Mann–Whitney U test for nonparametric continuous variables. Missing values were imputed by multiple imputations with five repetitions for handling.

We performed a propensity score matching analysis to achieve a more balanced comparison between the two groups. Propensity scores were calculated using a multivariate logistic regression model that included the following baseline covariates: age, sex, smoking status, alcohol intake, the presence of hypertension, diabetes mellitus, coronary artery disease (CAD), heart failure, peripheral artery disease, cerebrovascular accident and malignancy; levels of albumin, blood urea nitrogen, hemoglobin, hemoglobin A1c, eGFR, sodium, potassium, calcium, phosphate, high-density lipoprotein cholesterol, low-density lipoprotein cholesterol, triglycerides and bicarbonate; urine protein-to-creatinine ratio; and the use of renin–angiotensin system inhibitors (RASi), beta-blockers, calcium channel blockers (CCBs), sodium-glucose cotransporter 2 inhibitors (SGLT2i), glucagon-like peptide-1 receptor agonists and diuretics (excluding MRAs). Each spironolactone user was matched to two nonusers using nearest-neighbor matching without replacement based on their propensity scores.

Kaplan–Meier survival analysis was utilized to evaluate the time to each outcome, including ESRD, MACE, all-cause mortality and severe hyperkalemia, with statistical significance estimated using the log-rank test. Cox proportional hazards regression models were utilized to assess the relative risks of outcomes between spironolactone users and nonusers. The Cox models were adjusted for covariates with a standardized mean difference (SMD) >0.1 to account for potential confounders after propensity score matching. Additionally, to account for mortality as a competing event for ESRD and MACE, we performed competing risk analyses using both cause-specific hazard models and Fine–Gray subdistribution hazard models. Subgroup analyses were performed to evaluate interactions between spironolactone use and various baseline characteristics, including age, sex, baseline eGFR and comorbidities. Interaction effects were examined using likelihood ratio tests, and the corresponding hazard ratios with 95% confidence intervals for each subgroup were illustrated in a forest plot.

A sensitivity analysis was performed using inverse probability of treatment weighting (IPTW). The propensity score model included age, hypertension, coronary artery disease, heart failure, cancer, albumin, blood urea nitrogen, low-density lipoprotein, triglycerides, hemoglobin and the use of RASi, SGLT2i, beta-blockers, CCBs and diuretics. IPTW-adjusted Cox proportional hazards models were used to estimate adjusted hazard ratios (aHRs), with additional adjustment for covariates that remained imbalanced after weighting (SMD >0.1).

Statistical Package for the Social Sciences software (version 24) for Windows (IBM Company, Chicago, USA) and R software (version 4.4.3) for Windows were used for all statistical analyses. A two-tailed *P* < .05 was considered statistically significant.

## RESULTS

A total of 16 457 patients with CKD met the inclusion criteria. Of these patients, 2711 patients were spironolactone users, and 13 746 patients were nonusers. After propensity score matching, 2711 spironolactone users and 5422 nonusers were included in the analysis. The median age of the patients was 81.0 years (IQR 73.0–87.0 years), and 37.4% of the patients were female. The median follow-up time in the study cohort was 3.41 years (IQR 1.71–5.71 years). Table 1 presents the baseline characteristics of the patients.

**Table 1:** Baseline characteristics of the study population.

	Before propensity score matching				After propensity score matching			
	All patients	Spironolactone users	Spironolactone nonusers	SMD	All patients	Spironolactone users	Spironolactone nonusers	SMD
	(*n* = 16457)	(*n* = 2711)	(*n* = 13746)		(*n* = 8133)	(*n* = 2711)	(*n* = 5422)	
Age, years	79.0 (70.0, 86.0)	82.0 (73.0, 87.0)	79.0 (70.0, 86.0)	0.181	81.0 (73.0, 87.0)	82.0 (73.0, 87.0)	81.0 (72.0, 87.0)	0.040
Female sex, *n* (%)	6159 (37.4)	1010 (37.3)	5149 (37.5)	0.004	3038 (37.4)	1010 (37.3)	2028 (37.4)	0.003
Smokers, *n* (%)	658 (4.0)	97 (3.6)	561 (4.1)	0.026	322 (4.0)	97 (3.6)	225 (4.1)	0.030
Alcohol intake, *n* (%)	440 (2.7)	80 (3.0)	360 (2.6)	0.020	232 (2.9)	80 (3.0)	152 (2.8)	0.009
Hypertension, *n* (%)	12 444 (75.6)	2277 (84.0)	10 167 (74.0)	0.248	6826 (83.9)	2277 (84.0)	4549 (83.9)	0.003
Diabetes mellitus, *n* (%)	5240 (31.8)	939 (34.6)	4301 (31.3)	0.071	2839 (34.9)	939 (34.6)	1900 (35.0)	0.009
CAD, *n* (%)	3912 (23.8)	1051 (38.8)	2861 (20.8)	0.400	2863 (35.2)	1051 (38.8)	1812 (33.4)	0.112
Heart failure, *n* (%)	1728 (10.5)	818 (30.2)	910 (6.6)	0.638	1656 (20.4)	818 (30.2)	838 (15.5)	0.356
PAD, *n* (%)	49 (0.3)	13 (0.5)	36 (0.3)	0.036	38 (0.5)	13 (0.5)	25 (0.5)	0.003
CVA, *n* (%)	3143 (19.1)	538 (19.8)	2605 (19.0)	0.023	1648 (20.3)	538 (19.8)	1110 (20.5)	0.016
Malignancy, *n* (%)	4924 (29.9)	667 (24.6)	4257 (31.0)	0.142	2048 (25.2)	667 (24.6)	1381 (25.5)	0.020
Albumin, mg/dL	4.0 (3.5, 4.3)	3.9 (3.4, 4.2)	4.0 (3.6, 4.3)	0.151	3.9 (3.5, 4.3)	3.9 (3.4, 4.2)	3.9 (3.5, 4.3)	0.062
BUN, mg/dL	22.0 (18.0, 28.0)	23.0 (18.0, 30.0)	22.0 (18.0, 27.0)	0.157	22.0 (18.0, 29.0)	23.0 (18.0, 30.0)	22.0 (18.0, 28.0)	0.113
Hgb, g/dL	12.2 (10.7, 13.5)	11.9 (10.5, 13.2)	12.2 (10.8, 13.5)	0.137	12.0 (10.5, 13.3)	11.9 (10.5, 13.2)	12.0 (10.6, 13.3)	0.059
HbA_1c_, %	6.5 (5.9, 7.4)	6.4 (5.9, 7.3)	6.5 (5.9, 7.4)	0.054	6.5 (5.9, 7.4)	6.4 (5.9, 7.3)	6.5 (5.9, 7.4)	0.029
eGFR, mL/min/1.73 m^2^	53.3 (47.7, 57.0)	52.7 (47.0, 56.6)	53.4 (47.7, 57.0)	0.031	52.9 (47.4, 56.7)	52.7 (47.1, 56.6)	53.1 (47.4, 56.8)	0.020
30–59	15 978 (97.1)	2644 (97.5)	13 334 (97.0)		7924 (97.4)	2644 (97.5)	5280 (97.4)	
15–29	370 (2.2)	61 (2.3)	309 (2.2)		175 (2.2)	61 (2.3)	114 (2.1)	
<15	109 (0.7)	6 (0.2)	103 (0.8)		34 (0.4)	6 (0.2)	28 (0.5)	
Na, mmol/L	140.0 (137.0, 142.0)	140.0 (137.0, 142.0)	140.0 (137.0, 142.0)	0.094	140.0 (137.0, 142.0)	140.0 (137.0, 142.0)	140.0 (137.0, 142.0)	0.039
K, mmol/L	4.1 (3.8, 4.5)	4.1 (3.8, 4.5)	4.1 (3.8, 4.5)	0.021	4.1 (3.8, 4.5)	4.1 (3.8, 4.5)	4.1 (3.7, 4.4)	0.008
Ca, mg/dL	9.1 (8.6, 9.5)	9.0 (8.6, 9.5)	9.1 (8.6, 9.5)	0.078	9.0 (8.6, 9.5)	9.0 (8.6, 9.5)	9.1 (8.6, 9.5)	0.039
Phosphate, mg/dL	3.3 (2.8, 3.7)	3.2 (2.8, 3.7)	3.3 (2.8, 3.7)	0.027	3.3 (2.8, 3.7)	3.2 (2.8, 3.7)	3.3 (2.8, 3.7)	0.012
HDL cholesterol, mg/dL	43.0 (36.0, 53.0)	43.0 (35.0, 53.0)	44.0 (36.0, 53.0)	0.065	43.0 (36.0, 53.0)	43.0 (35.0, 53.0)	43.0 (36.0, 53.0)	0.034
LDL cholesterol, mg/dL	93.0 (74.0, 115.0)	90.0 (71.0, 112.0)	94.0 (74.0, 116.0)	0.114	90.0 (72.0, 112.0)	90.0 (71.0, 112.0)	91.0 (73.0, 112.0)	0.033
TG, mg/dL	105.0 (77.0, 149.0)	113.0 (82.0, 159.0)	112.0 (81.0, 157.0)	0.127	108.0 (79.0, 152.0)	105.0 (77.0, 149.0)	109.0 (79.0, 153.0)	0.065
Bicarbonate, mmol/L	24.0 (20.9, 27.6)	24.2 (21.1, 27.9)	23.9 (20.9, 27.6)	0.041	24.0 (21.0, 27.7)	24.2 (21.1, 27.9)	23.3 (20.9, 27.6)	0.034
UPCR, g/g	0.1 (0.0, 0.5)	0.2 (0.1, 0.6)	0.1 (0.0, 0.5)	0.007	0.2 (0.0, 0.6)	0.1 (0.1, 0.6)	0.1 (0.0, 0.5)	0.002
RASi, *n* (%)	9075 (55.1)	2076 (76.6)	6999 (50.9)	0.554	6014 (73.9)	2076 (76.6)	3938 (72.6)	0.091
Beta blockers, *n* (%)	6965 (42.3)	1828 (67.4)	5137 (37.4)	0.631	5175 (63.6)	1828 (67.4)	3347 (61.7)	0.119
CCBs, *n* (%)	9905 (60.2)	1996 (73.6)	7909 (57.5)	0.344	6047 (74.4)	1996 (73.6)	4051 (74.7)	0.025
SGLT2is, *n* (%)	857 (5.2)	242 (8.9)	615 (4.5)	0.179	670 (8.2)	242 (8.9)	428 (7.9)	0.037
GLP1RAs, *n* (%)	137 (0.8)	35 (1.3)	102 (0.7)	0.055	97 (1.2)	35 (1.3)	62 (1.1)	0.013
Diuretics, *n* (%)	5570 (33.8)	2137 (78.8)	3433 (25.0)	1.279	5558 (68.3)	2137 (78.8)	3421 (63.1)	0.352

Data are presented as *n* (%) or median and interquartile range.

BUN, blood urea nitrogen; Ca, calcium; CVA, cerebrovascular accident; GLP1RAs, glucagon-like peptide-1 receptor agonists; HbA1c, hemoglobin A1c; HDL, high-density lipoprotein; Hgb, hemoglobin; K, potassium; LDL, low-density lipoprotein; Na, sodium; PAD, peripheral artery disease; TG, triglycerides; UPCR, urine protein-to-creatinine ratio.

During the follow-up period, spironolactone users experienced a higher rate of all-cause mortality (5.70 versus 4.09 per 100 person-years) and hyperkalemia (3.09 versus 1.77 per 100 person-years). In a multivariate Cox regression analysis adjusted for potential confounders, the spironolactone users exhibited higher risks of all-cause mortality [aHR 1.23; 95% confidence interval (CI) 1.11–1.37; *P* < .001] and severe hyperkalemia (aHR 1.44; 95% CI 1.24–1.68; *P* < .001). However, regarding cardiovascular outcomes, spironolactone users exhibited a lower risk of MACE (aHR 0.90; 95% CI 0.82–0.99; *P* = .037), primarily due to a significant reduction in stroke risk (aHR 0.79; 95% CI 0.71–0.88; *P* < .001). Further analysis of stroke subtypes showed that this association was mainly driven by a reduction in ischemic stroke (aHR 0.77; 95% CI 0.68–0.86; *P* < .001), with no significant difference observed for hemorrhagic stroke (Supplementary data, Table S1). The incidence of ESRD was 1.08 per 100 person-years among spironolactone users, compared with 0.87 per 100 person-years in nonusers. After adjusting for relevant covariates, the risk of ESRD did not differ significantly between the spironolactone users and nonusers (aHR 1.09; 95% CI 0.85–1.38; *P* = .507) (Table 2). To further account for mortality as a competing event, we performed competing risk analyses for ESRD and MACE using both cause-specific and Fine–Gray subdistribution hazard models. These competing risk models yielded results consistent with the Cox model, showing no significant difference in ESRD risk between spironolactone users and nonusers. They also confirmed the reduction in MACE risk, which was largely driven by fewer stroke events (Table 3).

**Table 2:** Risks of ESRD, MACE, all-cause mortality and severe hyperkalemia between spironolactone users and matched nonusers.

	Spironolactone users			Spironolactone nonusers						
Outcomes	Number of events	Person-years	Incidence rate^a^	Number of events	Person-years	Incidence rate^a^	HR (95% CI)	*P*	Adjusted HR^b^ (95% CI)	*P*
ESRD^c^	113	10 483	1.08	182	20 898	0.87	1.23 (0.97–1.56)	.081	1.09 (0.85–1.38)	.507
MACE	663	8620	7.69	1341	17 089	7.85	0.98 (0.89–1.08)	.678	0.90 (0.82–0.99)	.037
Myocardial infarction	192	10 268	1.87	227	20 855	1.09	1.71 (1.41–2.07)	<.001	1.51 (1.23–1.84)	<.001
Stroke	507	9039	5.61	1172	17 582	6.67	0.85 (0.76–0.94)	.002	0.79 (0.71–0.88)	<.001
Cardiovascular death	178	10 758	1.65	278	21 447	1.30	1.27 (1.06–1.54)	.011	1.02 (0.84–1.24)	.820
All-cause mortality	613	10 758	5.70	878	21 447	4.09	1.39 (1.25–1.54)	<.001	1.23 (1.11–1.37)	<.001
Severe hyperkalemia	329	10 656	3.09	381	21 468	1.77	1.75 (1.51–2.03)	<.001	1.44 (1.24–1.68)	<.001

a Per 10^2^ person-years.

b Adjusted for CAD, heart failure, BUN, beta blockers and diuretics after propensity-score matching.

c ESRD was defined as initiation of long-term dialysis or kidney transplantation.

BUN, blood urea nitrogen.

**Table 3:** Competing risks analysis comparing spironolactone users and matched nonusers.

	Cause-specific hazard model		Fine–Gray subdistribution hazard model	
	cHR (95% CI)	*P*	sHR (95% CI)	*P*
Crude				
ESRD^b^	1.23 (0.97–1.56)	.081	1.20 (0.95–1.51)	.130
MACE	0.98 (0.89–1.08)	.675	0.96 (0.88–1.05)	.410
Myocardial infarction	1.71 (1.40–2.07)	<.001	1.67 (1.38–2.03)	<.001
Stroke	0.85 (0.76–0.94)	<.001	0.83 (0.75–0.92)	<.001
Cardiovascular death	1.27 (1.06–1.54)	.012	1.22 (1.01–1.47)	.040
Adjusted^a^				
ESRD^b^	1.09 (0.85–1.38)	.507	1.06 (0.84–1.35)	.630
MACE	0.90 (0.82–0.99)	<.001	0.89 (0.81–0.98)	.022
Myocardial infarction	1.51 (1.23–1.84)	<.001	1.49 (1.22–1.82)	<.001
Stroke	0.79 (0.71–0.88)	<.001	0.78 (0.70–0.87)	<.001
Cardiovascular death	1.02 (0.84–1.24)	.823	0.99 (0.82–1.21)	.930

a Adjusted for CAD, heart failure, BUN, beta blockers and diuretics after propensity-score matching.

b ESRD was defined as initiation of long-term dialysis or kidney transplantation.

BUN, blood urea nitrogen; cHR, cause-specific hazard ratio; sHR, subdistribution hazard ratio.

**Figure 1:**
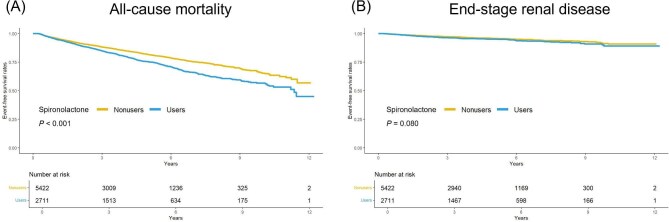
Kaplan–Meier curves for the risk of (**A**) all-cause mortality and (**B**) ESRD in spironolactone users versus nonusers. The event-free survival curves with the log-rank test indicate that spironolactone users exhibited a significantly higher risk of all-cause mortality than nonusers (*P* < .001) as indicated in panel (A). Panel (B) indicates no statistically significant difference in the risk of ESRD between the two groups (*P* = .080).

Figure 1 depicts the results of the Kaplan–Meier survival analysis log-rank test for all-cause mortality and ESRD. Spironolactone users exhibited significantly higher risks of all-cause mortality (log-rank test, *P <* .001) than the nonusers. However, the risk of developing ESRD exhibited no significant difference between the two groups (log-rank test, *P* = .080) during the follow-up period.

Analysis of primary causes of death showed that infection was the leading cause in both spironolactone users (40.8%) and nonusers (39.3%), followed by cardiovascular disease and cancer (Supplementary data, Table S2). In Cox regression models, spironolactone use was associated with significantly higher risks of non-cardiovascular mortality (aHR 1.33; 95% CI 1.17–1.52; *P* < .001) and infection-related mortality (aHR 1.26; 95% CI 1.07–1.49; *P* = .007). Cancer-related mortality did not differ significantly between groups (aHR 1.15; 95% CI 0.82–1.61; *P* = .415) (Supplementary data, Table S3).

**Figure 2:**
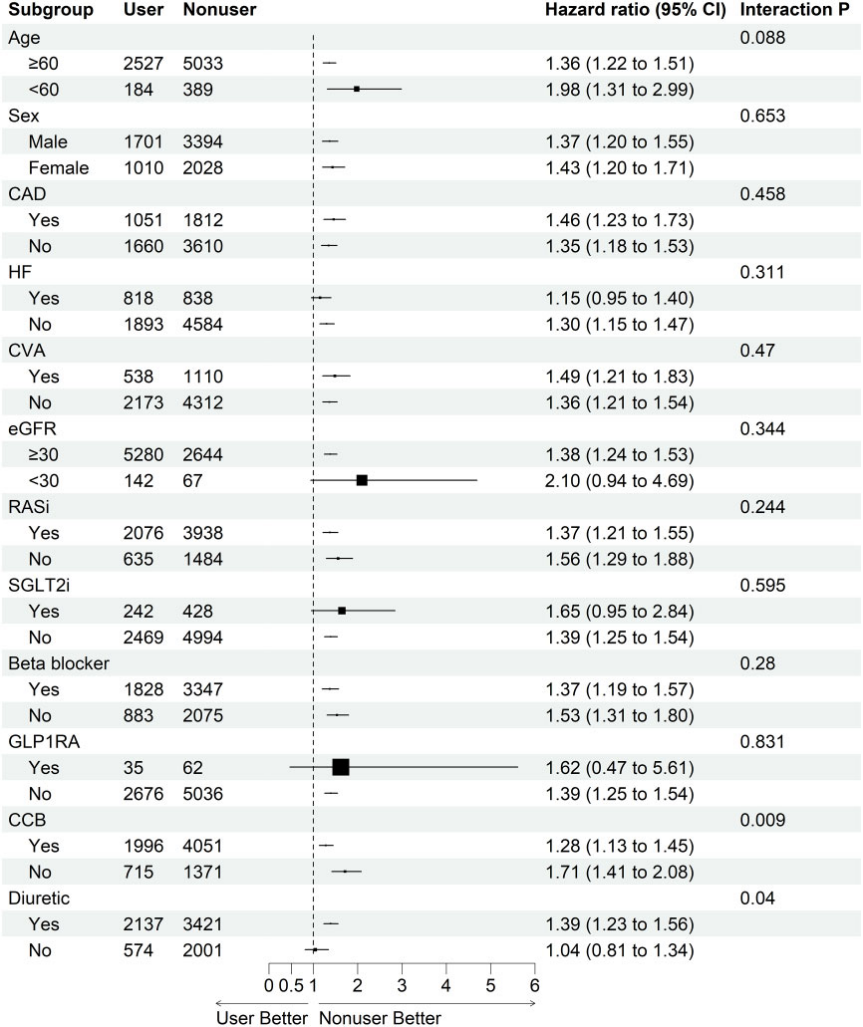
Subgroup analysis of the effects of spironolactone on all-cause mortality risk in patients with CKD. HTN, hypertension; DM, diabetes mellitus; HF, heart failure; CVA, cerebrovascular accident; GLP1RA, glucagon-like peptide-1 receptor agonists.

The subgroup analysis revealed that the risk of all-cause mortality was significantly higher among spironolactone users than among nonusers across most subgroups (Fig. 2). Significant interactions were observed for patients with hypertension (*P* for interaction = .026), CCB use (*P* for interaction = .009), and diuretic use (*P* for interaction = .040). Specifically, among non-hypertensive patients, spironolactone users exhibited a higher risk of mortality than nonusers (HR 1.80; 95% CI 1.40–2.30), while among patients with hypertension, the corresponding HR was 1.32 (95% CI 1.18–1.48). In patients who were not using CCBs, spironolactone users exhibited a significantly higher risk of mortality compared with nonusers (HR 1.71; 95% CI 1.41–2.08), while in those using CCBs, the HR was 1.28 (95% CI 1.13–1.45). Among diuretic users, the mortality risk was higher for spironolactone users than nonusers (HR 1.39; 95% CI 1.23–1.56), while in nonusers of diuretics, the HR was 1.04 (95% CI 0.81–1.34).

**Figure 3:**
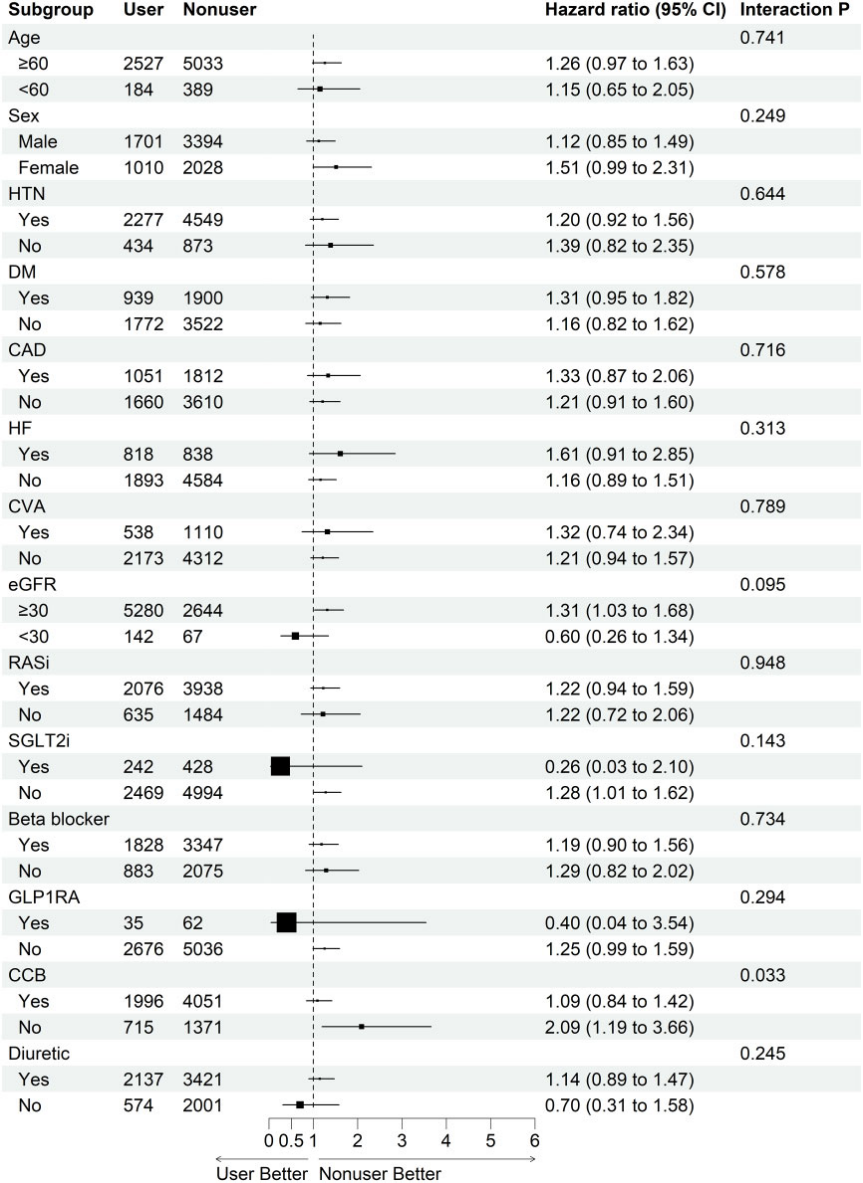
Subgroup analysis of the effects of spironolactone ESRD risk in patients with CKD. HTN, hypertension; DM, diabetes mellitus; HF, heart failure; CVA, cerebrovascular accident; GLP1RA, glucagon-like peptide-1 receptor agonists.

Subgroup analyses of ESRD risk revealed a significant interaction with CCB use (*P* for interaction = .033) (Fig. 3). Among patients not receiving CCBs, spironolactone users exhibited a higher risk of ESRD than nonusers (HR 2.09; 95% CI 1.19–3.66), while in CCB users, the HR was 1.09 (95% CI 0.84–1.42).

To further evaluate the relationship between spironolactone exposure duration and outcomes, we stratified patients based on the proportion of follow-up time during which spironolactone was used. In Cox regression analysis, patients who received spironolactone for >75% of the follow-up time had significantly increased risks of all-cause mortality (aHR 5.13; 95% CI 3.70–7.11; *P* < .001) and severe hyperkalemia (aHR 3.90; 95% CI 2.24–6.79; *P* < .001) compared with those with ≤75% exposure or nonusers. Conversely, these high-exposure patients appeared to have lower risks of MACE (aHR 0.55; 95% CI 0.28–1.11; *P* = .097) and stroke (aHR 0.50; 95% CI 0.22–1.12; *P* = .092), although neither result reached statistical significance. The full results are presented in Supplementary data, Table S4. Given the limited number of long-term users, we additionally examined the dose–response relationship by evaluating cumulative spironolactone dose and daily mean dose throughout the follow-up period. Patients in the highest cumulative dose tertile had significantly higher risks of all-cause mortality (aHR 1.17; 95% CI 1.01–1.35; *P* = .036) and severe hyperkalemia (aHR 1.43; 95% CI 1.18–1.74; *P* < .001), but lower risks of MACE (aHR 0.80; 95% CI 0.69–0.93; *P* = .003) and stroke (aHR 0.77; 95% CI 0.66–0.90; *P* = .001) (Supplementary data, Table S5). Similarly, patients with daily mean doses >50 mg had the highest risks of all-cause mortality (aHR 3.91; 95% CI 3.01–5.07; *P* < .001) and severe hyperkalemia (aHR 3.35; 95% CI 2.20–5.12; *P* < .001), along with significantly lower risks of MACE (aHR 0.60; 95% CI 0.37–0.97; *P* = .038) and stroke (aHR 0.56; 95% CI 0.33–0.94; *P* = .029) (Supplementary data, Table S6).

Among patients with severe hyperkalemia, post-event management strategies and their associations with clinical outcomes were evaluated. Of these 710 patients, 468 (65.9%) received either potassium binders or a modification in spironolactone therapy. The overall recurrence rate of hyperkalemia was 15.8%, with the lowest observed in patients who discontinued spironolactone (5.3%), followed by those who received dose reduction (11.6%) and potassium binders alone (19.6%) (Supplementary data, Table S7). In Cox regression analyses, patients who received both potassium binders and spironolactone adjustment had significantly higher risks of myocardial infarction (aHR 2.23; 95% CI 1.20–4.16; *P* = .011) and all-cause mortality (aHR 1.52; 95% CI 1.12–2.06; *P* = .007), compared with those who received no management. No significant differences were observed in ESRD or stroke risk across groups (Supplementary data, Table S8).

To evaluate the robustness of the main findings, we conducted a sensitivity analysis using IPTW. The propensity score model included age, hypertension, coronary artery disease, heart failure, cancer, albumin, blood urea nitrogen, low-density lipoprotein, triglycerides, hemoglobin, and the use of RASi, SGLT2i, beta-blockers, CCBs and diuretics. Most baseline characteristics were adequately balanced after weighting. However, some residual imbalance remained in age, hypertension, and use of RASi, beta-blockers and CCBs, with SMDs >0.1 (Supplementary data, Table S9). IPTW-adjusted Cox regression models, with additional adjustment for age, hypertension, RASi, beta-blockers and CCBs, demonstrated results consistent with the primary analysis. Spironolactone use was associated with a significantly lower risk of MACE (aHR 0.85; 95% CI 0.73–0.97; *P* < .001) and stroke (aHR 0.80; 95% CI 0.69–0.94; *P* = .006), but a higher risk of all-cause mortality (aHR 1.36; 95% CI 1.17–1.59; *P* < .001) and severe hyperkalemia (aHR 1.44; 95% CI 1.24–1.68; *P* < .001). The risk of ESRD remained nonsignificant (aHR 0.90; 95% CI 0.67–1.21; *P* = .485) (Supplementary data, Table S10).

## DISCUSSION

This retrospective study demonstrated that spironolactone administration in patients with CKD was associated with a reduced risk of MACE, primarily due to a lower incidence of stroke, and increased risks of all-cause mortality and severe hyperkalemia. The progression to ESRD showed no significant difference between spironolactone users and nonusers. Furthermore, our dose–response analyses provide additional insights into the clinical impact of spironolactone. Both higher cumulative exposure and greater daily mean doses were independently associated with increased risks of mortality and severe hyperkalemia. Notably, stroke risk was consistently reduced across different dose categories, with the most substantial reduction observed among patients receiving daily doses >50 mg. This combination of cerebrovascular benefit and increased metabolic or mortality risks reflects the trade-offs involved in spironolactone treatment in CKD, supporting the need for cautious patient selection, appropriate dose adjustment and individualized clinical decision-making.

Our findings are consistent with those of previous studies that have demonstrated the cerebrovascular benefits of MRAs, particularly in reducing ischemic stroke risk. Hyperaldosteronism and the inappropriate activation of mineralocorticoid receptors are associated with endothelial dysfunction, vascular remodeling and increased vascular stiffness, all of which predispose patients to cerebrovascular events [23–25]. Experimental models indicate that spironolactone enhances the structure and tone of cerebral vessels, consequently mitigating the severity of ischemic infarction​. These effects are probably mediated by spironolactone's ability to reduce oxidative stress, inflammation, and fibrosis, independent of blood pressure reduction [26, 27].

The dose-dependent relationship between spironolactone use and stroke reduction observed in another study reinforces the potential of MRAs to mitigate cerebrovascular risk in patients with hypertension [28].

While our study demonstrated the potential cardiovascular benefits of spironolactone, the increased risks of severe hyperkalemia and all-cause mortality among spironolactone users require careful evaluation in clinical practice. Hyperkalemia is a well-documented complication of MRA therapy in patients with CKD. Previous studies have demonstrated that the risk of hyperkalemia increases in patients with low eGFR, particularly with higher doses of spironolactone or the concurrent use of angiotensin-converting enzyme inhibitors and angiotensin receptor blockers [29, 30]. Severe hyperkalemia also significantly increases the risk of mortality in patients with CKD, as well established in prior studies [31, 32]. In our study, although several strategies were employed to manage hyperkalemia, patients requiring both potassium binders and spironolactone adjustment experienced poorer outcomes, which may reflect a higher burden of comorbidities and greater clinical complexity. Close monitoring, individualized dose titration, and dietary counseling remain crucial for minimizing the risk of hyperkalemia-related complications.

Besides hyperkalemia, the higher all-cause mortality observed among spironolactone users in this study may be attributed to several factors. Although spironolactone was associated with a reduced risk of MACE, it was also linked to a higher proportion of deaths from non-cardiovascular causes, especially infections. While propensity score matching was applied to minimize confounding, the retrospective nature of our study limits the ability to fully adjust for unmeasured variables such as frailty, nutritional status or undiagnosed immune dysfunction. These factors may have contributed to an increased vulnerability to infection-related mortality and should be considered when interpreting the results. In addition to these potential residual confounders, several pathophysiological mechanisms may also explain the observed associations. First, spironolactone users exhibited a higher prevalence of pre-existing heart failure and CAD. Second, spironolactone may impair the renal excretion of hydrogen ions, potentially exacerbating metabolic acidosis in patients with CKD [32]. This condition is associated with increased mortality owing to its detrimental effects on muscle wasting, bone health and cardiovascular function [33–35]. Third, spironolactone acts as an aldosterone antagonist, which reduces sodium reabsorption in the late distal tubule and collecting duct of the nephron, potentially causing hyponatremia or exacerbating volume depletion in certain patients [36, 37]. Hyponatremia has been independently associated with adverse cardiovascular outcome [38, 39]. The combination of volume depletion and impaired sodium handling in susceptible patients with CKD may exacerbate hemodynamic stability, thereby increasing mortality [40, 41]. Although no significant differences in serum bicarbonate or sodium levels were observed between spironolactone users and nonusers, minor or transient imbalances may have remained undetected. The retrospective design of our analysis also limits the ability to capture short-term electrolyte disturbances that could have clinical relevance. Moreover, subgroup analysis revealed that spironolactone users who also received other diuretics had a significantly higher mortality rate, suggesting an additive or synergistic effect on fluid and electrolyte imbalance.

This study has some limitations. First, its retrospective design may have introduced selection bias and residual confounding despite the use of propensity score matching. Second, reliance on electronic health records might have led to incomplete data capture, particularly regarding medication adherence and lifestyle factors. Third, although left ventricular ejection fraction is a clinically important variable, it was not available in this study. Finally, the cohort was derived from a single tertiary center in Taiwan and had a relatively high median age, which may limit the generalizability of our findings to younger populations or non-Asian settings.

In conclusion, spironolactone use in patients with CKD was associated with a reduced risk of cardiovascular events, particularly stroke, but increased risks of all-cause mortality and severe hyperkalemia, without a significant effect on ESRD. Individualized patient selection, appropriate dose adjustment and careful monitoring are important to balance the potential benefits and mitigate adverse effects of spironolactone therapy.

## Supplementary Material

sfaf247_Supplemental_File

## Data Availability

The data underlying this article will be shared on reasonable request to the corresponding author.
