# Supplementary material for: Effect of spironolactone on cardiovascular and renal outcomes in patients with chronic kidney disease
Source: Clin Kidney J. 2025 Aug 6;18(9):sfaf247. doi: 10.1093/ckj/sfaf247 (PMC12409270; doi:10.1093/ckj/sfaf247)
Supplement: sfaf247_Supplemental_File [file sfaf247_supplemental_file.docx]

**Effect of Spironolactone on Cardiovascular and Renal Outcomes in Patients with Chronic Kidney Disease**

**Authors:** Tz-Heng Chen, MD, Shuo-Ming Ou, MD, PhD, Kuan-Hsun Lin, Yang Ho, MD, Wei-Cheng Tseng, MD, PhD, Yuan-Chia Chu, PhD, Der-Cherng Tarng, MD, PhD

**Table of Contents**

| **Supplemental Tables** |  |
| --- | --- |
| Supplementary Table S1. Risks of ischemic and hemorrhagic Stroke between spironolactone users and matched nonusers | 3 |
| Supplementary Table S2. Distribution of primary causes of death among spironolactone users and nonusers | 4 |
| Supplementary Table S3. Risks of non-cardiovascular, infection-related, and cancer-related mortality between spironolactone users and matched nonusers | 5 |
| Supplementary Table S4. Risks of end-stage renal disease, major adverse cardiovascular events, all-cause mortality, and severe hyperkalemia according to spironolactone use >75% or ≤75% of follow-up time | 6 |
| Supplementary Table S5. Risks of end-stage renal disease, major adverse cardiovascular events, all-cause mortality, and severe hyperkalemia according to cumulative spironolactone dose tertiles | 7-9 |
| Supplementary Table S6. Risks of end-stage renal disease, major adverse cardiovascular events, all-cause mortality, and severe hyperkalemia according to mean daily spironolactone dose | 10-11 |
| Supplementary Table S7. Management strategies for severe hyperkalemia and associated recurrence rates | 12 |
| Supplementary Table S8. Risks of end-stage renal disease, major adverse cardiovascular events, and all-cause mortality according to management strategies in patients with severe hyperkalemia | 13-14 |
| Supplementary Table S9. Baseline characteristics between spironolactone users and nonusers before and after inverse probability of treatment weighting | 15-16 |
| Supplementary Table S10. Risks of end-stage renal disease, major adverse cardiovascular events, all-cause mortality, and severe hyperkalemia between spironolactone users and nonusers in the IPTW-adjusted cohort | 17 |

**Supplementary Tables**

**Supplementary Table S1. Risks of ischemic and hemorrhagic Stroke between spironolactone users and matched nonusers**

|  | **Spironolactone users** | | | **Spironolactone nonusers** | | |  |  |  |  |
| --- | --- | --- | --- | --- | --- | --- | --- | --- | --- | --- |
| **Outcomes** | **Number of events** | **Person-years** | **Incidence rate**^a^ | **Number of events** | **Person-years** | **Incidence rate**^a^ | **HR**  **(95% CI)** | **P** | **Adjusted HR**^b^  **(95% CI)** | **P** |
| **Ischemic stroke** | 444 | 9039 | 4.91 | 1,046 | 17582 | 5.95 | 0.83 (0.74－0.93) | 0.001 | 0.77 (0.68－0.86) | <0.001 |
| **Hemorrhagic stroke** | 63 | 9039 | 0.70 | 126 | 17582 | 0.72 | 0.98 (0.72－1.32) | 0.883 | 0.97 (0.71－1.32) | 0.838 |
| ^a^per 10^2^ person-years.  ^b^Adjusted for CAD, heart failure, BUN, beta blockers and diuretics after propensity-score matching.  Abbreviations: BUN, blood urea nitrogen; CAD, coronary artery disease; CI, confidence interval; HR, hazard ratio; MACE, major adverse cardiovascular events. | | | | | | | | | | |

**Supplementary Table S2. Distribution of primary causes of death among spironolactone users and nonusers**

|  | **All patients (n = 1490)** | **Spironolactone users**  **(n = 613)** | **Spironolactone nonusers**  **(n = 877)** | **P** |
| --- | --- | --- | --- | --- |
| **Primary cause of death** |  |  |  | 0.119 |
| **Infection** | 595 (39.9) | 250 (40.8) | 345 (39.3) |  |
| **Cardiovascular disease** | 456 (30.6) | 178 (29.0) | 278 (31.7) |  |
| **Cancer** | 156 (10.5) | 54 (8.8) | 102 (11.6) |  |
| **Lung disease** | 80 (5.4) | 37 (6.0) | 43 (4.9) |  |
| **Kidney failure** | 36 (2.4) | 21 (3.4) | 15 (1.7) |  |
| **Liver failure** | 3 (0.2) | 2 (0.3) | 1 (0.1) |  |
| **Other** | 164 (11.0) | 71 (11.6) | 93 (10.6) |  |
| Data are presented as n (%) |  |  |  |  |

**Supplementary Table S3. Risks of non-cardiovascular, infection-related, and cancer-related mortality between spironolactone users and matched nonusers**

|  | **Spironolactone users** | | | **Spironolactone nonusers** | | |  |  |  |  |
| --- | --- | --- | --- | --- | --- | --- | --- | --- | --- | --- |
| **Outcomes** | **Number of events** | **Person-years** | **Incidence rate**^a^ | **Number of events** | **Person-years** | **Incidence rate**^a^ | **HR**  **(95% CI)** | **P** | **Adjusted HR**^b^  **(95% CI)** | **P** |
| **Non-cardiovascular mortality** | 435 | 10758 | 4.04 | 599 | 21447 | 2.79 | 1.44 (1.28－1.64) | <0.001 | 1.33 (1.17－1.52) | <0.001 |
| **Infection-related mortality** | 250 | 10758 | 2.32 | 345 | 21447 | 1.61 | 1.44 (1.23－1.70) | <0.001 | 1.26 (1.07－1.49) | 0.007 |
| **Cancer-related mortality** | 54 | 10758 | 0.50 | 102 | 21447 | 0.48 | 1.06 (0.76－1.47) | 0.748 | 1.15 (0.82－1.61) | 0.415 |
| ^a^per 10^2^ person-years.  ^b^Adjusted for CAD, heart failure, BUN, beta blockers and diuretics after propensity-score matching.  Abbreviations: BUN, blood urea nitrogen; CAD, coronary artery disease; CI, confidence interval; HR, hazard ratio; MACE, major adverse cardiovascular events. | | | | | | | | | | |

**Supplementary Table S4. Risks of end-stage renal disease, major adverse cardiovascular events, all-cause mortality, and severe hyperkalemia according to spironolactone use >75% or ≤75% of follow-up time**

|  | **Spironolactone use for >75% of follow-up time (n=77)** | | | **Nonusers or use ≤75% of follow-up time (n=8056)** | | |  |  |  |  |
| --- | --- | --- | --- | --- | --- | --- | --- | --- | --- | --- |
| **Outcomes** | **Number of events** | **Person-years** | **Incidence rate**^a^ | **Number of events** | **Person-years** | **Incidence rate**^a^ | **HR**  **(95% CI)** | **P** | **Adjusted HR**^b^  **(95% CI)** | **P** |
| **End-stage renal disease**^c^ | 3 | 118 | 2.54 | 292 | 31263 | 0.93 | 2.61 (0.83－8.15) | 0.100 | 2.16 (0.69－6.77) | 0.187 |
| **MACE** | 8 | 117 | 6.83 | 1996 | 25591 | 7.80 | 0.57 (0.28－1.14) | 0.111 | 0.55 (0.28－1.11) | 0.097 |
| **Myocardial infarction** | 2 | 124 | 1.61 | 417 | 30998 | 1.35 | 0.80 (0.20－3.21) | 0.753 | 0.76 (0.19－3.05) | 0.696 |
| **Stroke** | 6 | 118 | 5.10 | 1673 | 26504 | 6.31 | 0.51 (0.23－1.15) | 0.105 | 0.50 (0.22－1.12) | 0.092 |
| **Cardiovascular death** | 4 | 125 | 3,21 | 452 | 32080 | 1.14 | 3.11 (1.16－8.36) | 0.024 | 2.32 (0.86－6.25) | 0.096 |
| **All-cause mortality** | 38 | 125 | 30.5 | 1452 | 32080 | 4.53 | 7.16 (5.18－9.90) | <0.001 | 5.13 (3.70－7.11) | <0.001 |
| **Severe hyperkalemia** | 13 | 153 | 8.49 | 697 | 31970 | 2.18 | 5.28 (3.04－9.16) | <0.001 | 3.90 (2.24－6.79) | <0.001 |
| ^a^per 10^2^ person-years.  ^b^Adjusted for CAD, heart failure, BUN, beta blockers and diuretics.  ^c^End-stage renal disease was defined as initiation of long-term dialysis or kidney transplantation.  Abbreviations: BUN, blood urea nitrogen; CAD, coronary artery disease; CI, confidence interval; HR, hazard ratio; MACE, major adverse cardiovascular events. | | | | | | | | | | |

**Supplementary Table S5. Risks of end-stage renal disease, major adverse cardiovascular events, all-cause mortality, and severe hyperkalemia according to cumulative spironolactone dose tertiles**

|  | **Number of subjects** | **Number of event (%)** | **Crude HR**  **(95% CI)** | **P** | **Adjusted HR**^a^  **(95% CI)** | **P** |
| --- | --- | --- | --- | --- | --- | --- |
| **End-stage renal disease**^b^ |  |  |  |  |  |  |
| Nonuser | 5422 | 182 (3.4) | Reference |  | Reference |  |
| Lower tertile^c^ | 925 | 24 (2.6) | 0.92 (0.60－1.40) | 0.683 | 0.83 (0.54－1.27) | 0.391 |
| Middle tertile | 883 | 41 (4.6) | 1.48 (1.06－2.08) | 0.022 | 1.34 (0.95－1.88) | 0.100 |
| Upper tertile | 903 | 48 (5.3) | 1.27 (0.92－1.74) | 0.143 | 1.08 (0.78－1.50) | 0.632 |
| **MACE** |  |  |  |  |  |  |
| Nonuser | 5422 | 1341 (24.7) | Reference |  | Reference |  |
| Lower tertile | 925 | 207 (22.4) | 0.98 (0.84－1.13) | 0.764 | 0.92 (0.79－1.07) | 0.269 |
| Middle tertile | 883 | 230 (26.0) | 1.11 (0.96－1.28) | 0.146 | 1.01 (0.87－1.16) | 0.910 |
| Upper tertile | 903 | 226 (25.0) | 0.88 (0.76－1.01) | 0.071 | 0.80 (0.69－0.93) | 0.003 |
| **Myocardial infarction** |  |  |  |  |  |  |
| Nonuser | 5422 | 227 (4.2) | Reference |  | Reference |  |
| Lower tertile | 925 | 51 (5.5) | 1.49 (1.10－2.02) | 0.010 | 1.34 (0.99－1.82) | 0.062 |
| Middle tertile | 883 | 85 (9.6) | 2.48 (1.93－3.18) | <0.001 | 2.12 (1.64－2.74) | <0.001 |
| Upper tertile | 903 | 56 (6.2) | 1.28 (0.96－1.72) | 0.097 | 1.13 (0.84－1.53) | 0.421 |
| **Stroke** |  |  |  |  |  |  |
| Nonuser | 5422 | 1172 (21.6) | Reference |  | Reference |  |
| Lower tertile | 925 | 163 (17.6) | 0.87 (0.74－1.03) | 0.104 | 0.83 (0.70－0.98) | 0.026 |
| Middle tertile | 883 | 155 (17.6) | 0.83 (0.70－0.98) | 0.031 | 0.77 (0.65－0.91) | 0.002 |
| Upper tertile | 903 | 189 (20.9) | 0.84 (0.72－0.98) | 0.024 | 0.77 (0.66－0.90) | 0.001 |
| **Cardiovascular death** |  |  |  |  |  |  |
| Nonuser | 5422 | 278 (5.1) | Reference |  | Reference |  |
| Lower tertile | 925 | 43 (4.6) | 1.16 (0.84－1.60) | 0.365 | 0.97 (0.70－1.34) | 0.854 |
| Middle tertile | 883 | 70 (7.9) | 1.69 (1.30－2.20) | <0.001 | 1.38 (1.06－1.81) | 0.017 |
| Upper tertile | 903 | 65 (7.2) | 1.06 (0.81－1.39) | 0.676 | 0.82 (0.62－1.08) | 0.151 |
| **All-cause mortality** |  |  |  |  |  |  |
| Nonuser | 5422 | 877 (16.2) | Reference |  | Reference |  |
| Lower tertile | 925 | 154 (16.6) | 1.25 (1.06－1.49) | 0.010 | 1.12 (0.94－1.33) | 0.206 |
| Middle tertile | 883 | 209 (23.7) | 1.58 (1.36－1.83) | <0.001 | 1.44 (1.23－1.68) | <0.001 |
| Upper tertile | 903 | 250 (27.7) | 1.35 (1.17－1.56) | <0.001 | 1.17 (1.01－1.35) | 0.036 |
| **Severe hyperkalemia** |  |  |  |  |  |  |
| Nonuser | 5422 | 381 (7.0) | Reference |  | Reference |  |
| Lower tertile | 925 | 75 (8.1) | 1.52 (1.19－1.95) | <0.001 | 1.30 (1.01－1.66) | 0.042 |
| Middle tertile | 883 | 104 (11.8) | 1.86 (1.50－2.31) | <0.001 | 1.59 (1.27－1.98) | <0.001 |
| Upper tertile | 903 | 150 (16.6) | 1.81 (1.50－2.19) | <0.001 | 1.43 (1.18－1.74) | <0.001 |
| ^a^Adjusted for CAD, heart failure, BUN, beta blockers and diuretics.  ^b^End-stage renal disease was defined as initiation of long-term dialysis or kidney transplantation.  ^c^Spironolactone users were grouped into tertiles according to their cumulative spironolactone dose (lower, <1050 mg; middle, 1050 U/ml to <5275 mg; upper, ≥5275 mg).  Abbreviations: BUN, blood urea nitrogen; CAD, coronary artery disease; CI, confidence interval; HR, hazard ratio; MACE, major adverse cardiovascular events. | | | | | | |

**Supplementary Table S6. Risks of end-stage renal disease, major adverse cardiovascular events, all-cause mortality, and severe hyperkalemia according to mean daily spironolactone dose**

|  | **Number of subjects** | **Number of event (%)** | **Crude HR**  **(95% CI)** | **P** | **Adjusted HR**^a^  **(95% CI)** | **P** |
| --- | --- | --- | --- | --- | --- | --- |
| **End-stage renal disease^b^** |  |  |  |  |  |  |
| Nonuser | 5422 | 182 (3.4) | Reference |  | Reference |  |
| ≤25 mg | 2113 | 80 (3.8) | 1.10 (0.84－1.43) | 0.481 | 0.99 (0.76－1.30) | 0.944 |
| >25 to ≤50 mg | 475 | 24 (5.1) | 1.52 (0.99－2.32) | 0.056 | 1.20 (0.78－1.85) | 0.413 |
| >50 mg | 123 | 9 (7.3) | 2.95 (1.51－5.76) | 0.002 | 2.50 (1.28－4.91) | 0.007 |
| **MACE** |  |  |  |  |  |  |
| Nonuser | 5422 | 1341 (24.7) | Reference |  | Reference |  |
| ≤25 mg | 2113 | 522 (24.7) | 0.99 (0.89－1.09) | 0.781 | 0.91 (0.82－1.01) | 0.083 |
| >25 to ≤50 mg | 475 | 124 (26.1) | 1.05 (0.88－1.26) | 0.587 | 0.93 (0.77－1.12) | 0.449 |
| >50 mg | 123 | 17 (13.8) | 0.59 (0.36－0.95) | 0.029 | 0.60 (0.37－0.97) | 0.038 |
| **Myocardial infarction** |  |  |  |  |  |  |
| Nonuser | 5422 | 227 (4.2) | Reference |  | Reference |  |
| ≤25 mg | 2113 | 147 (7.0) | 1.66 (1.35－2.05) | <0.001 | 1.47 (1.19－1.83) | <0.001 |
| >25 to ≤50 mg | 475 | 42 (8.8) | 2.15 (1.55－2.99) | <0.001 | 1.78 (1.27－2.50) | <0.001 |
| >50 mg | 123 | 3 (2.4) | 0.68 (0.22－2.13) | 0.510 | 0.81 (0.26－2.54) | 0.718 |
| **Stroke** |  |  |  |  |  |  |
| Nonuser | 5422 | 1172 (21.6) | Reference |  | Reference |  |
| ≤25 mg | 2113 | 403 (19.1) | 0.86 (0.77－0.96) | 0.008 | 0.80 (0.71－0.90) | <0.001 |
| >25 to ≤50 mg | 475 | 90 (18.9) | 0.86 (0.70－1.07) | 0.175 | 0.77 (0.62－0.96) | 0.021 |
| >50 mg | 123 | 14 (11.4) | 0.56 (0.33－0.95) | 0.033 | 0.56 (0.33－0.94) | 0.029 |
| **Cardiovascular death** |  |  |  |  |  |  |
| Nonuser | 5422 | 278 (5.1) | Reference |  | Reference |  |
| ≤25 mg | 2113 | 123 (5.8) | 1.10 (0.89－1.36) | 0.363 | 0.91 (0.73－1.13) | 0.380 |
| >25 to ≤50 mg | 475 | 48 (10.1) | 1.99 (1.47－2.71) | <0.001 | 1.43 (1.04－1.96) | 0.027 |
| >50 mg | 123 | 7 (5.7) | 1.66 (0.78－3.51) | 0.187 | 1.55 (0.73－3.29) | 0.252 |
| **All-cause mortality** |  |  |  |  |  |  |
| Nonuser | 5422 | 877 (16.2) | Reference |  | Reference |  |
| ≤25 mg | 2113 | 387 (18.3) | 1.11 (0.98－1.25) | 0.096 | 1.00 (0.88－1.13) | 0.957 |
| >25 to ≤50 mg | 475 | 165 (34.7) | 2.16 (1.83－2.55) | <0.001 | 1.75 (1.47－2.07) | <0.001 |
| >50 mg | 123 | 61 (49.6) | 4.20 (3.24－5.44) | <0.001 | 3.91 (3.01－5.07) | <0.001 |
| **Severe hyperkalemia** |  |  |  |  |  |  |
| Nonuser | 5422 | 381 (7.0) | Reference |  | Reference |  |
| ≤25 mg | 2113 | 226 (10.7) | 1.51 (1.28－1.78) | <0.001 | 1.28 (1.08－1.51) | 0.005 |
| >25 to ≤50 mg | 475 | 80 (16.8) | 2.49 (1.95－3.16) | <0.001 | 1.78 (1.39－2.28) | <0.001 |
| >50 mg | 123 | 23 (18.7) | 3.85 (2.53－5.87) | <0.001 | 3.35 (2.20－5.12) | <0.001 |
| ^a^Adjusted for CAD, heart failure, BUN, beta blockers and diuretics.  ^b^End-stage renal disease was defined as initiation of long-term dialysis or kidney transplantation.  Abbreviations: BUN, blood urea nitrogen; CAD, coronary artery disease; CI, confidence interval; HR, hazard ratio; MACE, major adverse cardiovascular events. | | | | | | |

**Supplementary Table S7. Management strategies for severe hyperkalemia and associated recurrence rates**

| **Management strategy**^a^ | **Number of Patients** | **Percentage (%)** | **Recurrent hyperkalemia**^b^ **(%)** |
| --- | --- | --- | --- |
| No management | 242 | 34.1 | 11.6 |
| Potassium binder only | 312 | 43.9 | 19.6 |
| Spironolactone dose reduction only | 6 | 0.8 | 16.7 |
| Spironolactone discontinuation^c^ only | 38 | 5.4 | 5.3 |
| Spironolactone adjustment^d^ only | 44 | 6.2 | 6.8 |
| Both potassium binder and spironolactone adjustment^d^ | 112 | 15.8 | 8.9 |
| Any management (potassium binder or spironolactone adjustment) | 468 | 65.9 | 15.8 |
| ^a^Management strategies were defined as any intervention initiated within 90 days after the initial hyperkalemia episode, including potassium binder use, spironolactone dose reduction, or spironolactone discontinuation. ^b^Recurrent hyperkalemia was defined as a serum potassium level >6.0 mmol/L occurring more than 90 days after the initial hyperkalemia episode. ^c^Spironolactone discontinuation was defined as permanent cessation of spironolactone during the study period. ^d^Spironolactone adjustment was defined as either dose reduction or permanent discontinuation of spironolactone following the initial hyperkalemia episode. | | | |

**Supplementary Table S8. Risks of end-stage renal disease, major adverse cardiovascular events, and all-cause mortality according to management strategies in patients with severe hyperkalemia**

|  | **Number of patients** | **Number of events**  **(%)** | **Crude HR**  **(95% CI)** | **P** | **Adjusted HR**^a^  **(95% CI)** | **P** |
| --- | --- | --- | --- | --- | --- | --- |
| **End-stage renal disease**^b^ |  |  |  |  |  |  |
| No management | 242 | 35 (14.5) | Reference |  | Reference |  |
| Potassium binder only | 312 | 53 (17.0) | 1.09 (0.71－1.67) | 0.696 | 1.01 (0.66－1.55) | 0.964 |
| Spironolactone dose adjustment only | 44 | 6 (13.6) | 1.24 (0.52－2.96) | 0.624 | 1.19 (0.50－2.85) | 0.696 |
| Both potassium binder and spironolactone adjustment | 112 | 12 (10.7) | 0.80 (0.41－1.54) | 0.505 | 0.81 (0.42－1.57) | 0.537 |
| **MACE** |  |  |  |  |  |  |
| No management | 242 | 79 (32.6) | Reference |  | Reference |  |
| Potassium binder only | 312 | 107 (34.3) | 1.01 (0.75－1.35) | 0.963 | 1.02 (0.76－1.37) | 0.899 |
| Spironolactone dose adjustment only | 44 | 11 (25.0) | 0.92 (0.49－1.72) | 0.787 | 0.91 (0.48－1.71) | 0.761 |
| Both potassium binder and spironolactone adjustment | 112 | 40 (35.7) | 1.15 (0.78－1.68) | 0.486 | 1.13 (0.76－1.66) | 0.550 |
| **Myocardial infarction** |  |  |  |  |  |  |
| No management | 242 | 21 (8.7) | Reference |  | Reference |  |
| Potassium binder only | 312 | 28 (9.0) | 1.01 (0.57－1.78) | 0.978 | 1.03 (0.59－1.82) | 0.909 |
| Spironolactone dose adjustment only | 44 | 4 (9.1) | 1.33 (0.46－3.87) | 0.604 | 1.35 (0.46－3.98) | 0.581 |
| Both potassium binder and spironolactone adjustment | 112 | 20 (17.9) | 2.23 (1.21－4.12) | 0.010 | 2.23 (1.20－4.16) | 0.011 |
| **Stroke** |  |  |  |  |  |  |
| No management | 242 | 69 (28.5) | Reference |  | Reference |  |
| Potassium binder only | 312 | 88 (28.2) | 0.94 (0.69－1.30) | 0.724 | 0.96 (0.70－1.32) | 0.799 |
| Spironolactone dose adjustment only | 44 | 7 (15.9) | 0.63 (0.29－1.37) | 0.239 | 0.62 (0.29－1.36) | 0.237 |
| Both potassium binder and spironolactone adjustment | 112 | 22 (19.6) | 0.72 (0.44－1.16) | 0.176 | 0.72 (0.44－1.17) | 0.180 |
| **Cardiovascular death** |  |  |  |  |  |  |
| No management | 242 | 46 (19.0) | Reference |  | Reference |  |
| Potassium binder only | 312 | 54 (17.3) | 0.89 (0.60－1.31) | 0.545 | 0.92 (0.62－1.37) | 0.687 |
| Spironolactone dose adjustment only | 44 | 4 (9.1) | 0.71 (0.26－1.98) | 0.514 | 0.81 (0.29－2.26) | 0.682 |
| Both potassium binder and spironolactone adjustment | 112 | 21 (18.8) | 1.25 (0.74－2.09) | 0.406 | 1.23 (0.73－2.08) | 0.442 |
| **All-cause mortality** |  |  |  |  |  |  |
| No management | 242 | 115 (47.5) | Reference |  | Reference |  |
| Potassium binder only | 312 | 152 (48.7) | 0.99 (0.78－1.26) | 0.933 | 1.03 (0.81－1.32) | 0.813 |
| Spironolactone dose adjustment only | 44 | 14 (31.8) | 0.93 (0.53－1.62) | 0.797 | 1.04 (0.59－1.82) | 0.894 |
| Both potassium binder and spironolactone adjustment | 112 | 68 (60.7) | 1.51 (1.12－2.04) | 0.0074 | 1.52 (1.12－2.06) | 0.007 |
| ^a^Adjusted for CAD, heart failure, BUN, beta blockers and diuretics.  ^b^End-stage renal disease was defined as initiation of long-term dialysis or kidney transplantation.  Abbreviations: BUN, blood urea nitrogen; CAD, coronary artery disease; CI, confidence interval; HR, hazard ratio; MACE, major adverse cardiovascular events. | | | | | | |

**Supplementary Table S9. Baseline characteristics between spironolactone users and nonusers before and after inverse probability of treatment weighting**

|  | **Before IPTW**^a^ | |  |  | **After IPTW**^b^ | |  |
| --- | --- | --- | --- | --- | --- | --- | --- |
|  | **Spironolactone  users** | **Spironolactone  nonusers** | **SMD** |  | **Spironolactone  users** | **Spironolactone  nonusers** | **SMD** |
|  | **(n =2711)** | **(n =13746)** |  |  | **(n =2711)** | **(n =13746)** |  |
| **Age, years** | 82.0 [73.0, 87.0] | 79.0 [70.0, 86.0] | 0.181 |  | 81.0 [73.0, 86.0] | 79.0 [70.0, 86.0] | 0.103 |
| **Female sex** | 1010 (37.3) | 5149 (37.5) | 0.004 |  | 37.3 | 37.5 | 0.004 |
| **Smokers** | 97 (3.6) | 561 (4.1) | 0.026 |  | 3.6 | 4.1 | 0.026 |
| **Alcohol intake** | 80 (3.0) | 360 (2.6) | 0.020 |  | 3.0 | 2.6 | 0.020 |
| **Hypertension** | 2277 (84.0) | 10167 (74.0) | 0.248 |  | 81.3 | 75.8 | 0.136 |
| **Diabetes mellitus** | 939 (34.6) | 4301 (31.3) | 0.071 |  | 34.6 | 31.3 | 0.071 |
| **CAD** | 1051 (38.8) | 2861 (20.8) | 0.400 |  | 27.1 | 24.0 | 0.072 |
| **Heart failure** | 818 (30.2) | 910 (6.6) | 0.638 |  | 11.5 | 10.6 | 0.027 |
| **PAD** | 13 (0.5) | 36 (0.3) | 0.036 |  | 0. | 0.3 | 0.036 |
| **CVA** | 538 (19.8) | 2605 (19.0) | 0.023 |  | 19.8 | 19.0 | 0.023 |
| **Malignancy** | 667 (24.6) | 4257 (31.0) | 0.142 |  | 28.9 | 30.1 | 0.027 |
| **Albumin, mg/dL** | 3.9 [3.4, 4.2] | 4.0 [3.6, 4.3] | 0.151 |  | 4.0 [3.5, 4.3] | 4.0 [3.5, 4.3] | 0.017 |
| **BUN, mg/dL** | 23.0 [18.0, 30.0] | 22.0 [18.0, 27.0] | 0.157 |  | 22.0 [18.0, 28.0] | 22.0 [18.0, 28.0] | 0.006 |
| **Hgb, g/dL** | 11.9 [10.5, 13.2] | 12.2 [10.8, 13.5] | 0.137 |  | 12.1 [10.8, 13.5] | 12.2 [10.7, 13.5] | 0.013 |
| **HbA_1c_, %** | 6.4 [5.9, 7.3] | 6.5 [5.9, 7.4] | 0.054 |  | 6.4 [5.9, 7.3] | 6.5 [5.9, 7.4] | 0.054 |
| **eGFR, mL/min/1.73 m^2^** | 52.7 [47.0, 56.6] | 53.4 [47.7, 57.0] | 0.031 |  | 52.7 [47.0, 56.6] | 53.4 [47.7, 57.0] | 0.031 |
| **Na, mmol/L** | 140.0 [137.0, 142.0] | 140.0 [137.0, 142.0] | 0.094 |  | 140.0 [137.0, 142.0] | 140.0 [137.0, 142.0] | 0.094 |
| **K, mmol/L** | 4.1 [3.8, 4.5] | 4.1 [3.8, 4.5] | 0.021 |  | 4.1 [3.8, 4.5] | 4.1 [3.8, 4.5] | 0.021 |
| **Ca, mg/dL** | 9.0 [8.6, 9.5] | 9.1 [8.6, 9.5] | 0.078 |  | 9.0 [8.6, 9.5] | 9.1 [8.6, 9.5] | 0.078 |
| **Phosphate, mg/dL** | 3.2 [2.8, 3.7] | 3.3 [2.8, 3.7] | 0.027 |  | 3.2 [2.8, 3.7] | 3.3 [2.8, 3.7] | 0.027 |
| **HDL cholesterol, mg/dL** | 43.0 [35.0, 53.0] | 44.0 [36.0, 53.0] | 0.065 |  | 43.0 [35.0, 53.0] | 44.0 [36.0, 53.0] | 0.065 |
| **LDL cholesterol, mg/dL** | 90.0 [71.0, 112.0] | 94.0 [74.0, 116.0] | 0.114 |  | 93.0 [72.0, 115.0] | 93.0 [74.0, 115.0] | 0.023 |
| **TG, mg/dL** | 113.0 [82.0, 159.0] | 112.0 [81.0, 157.0] | 0.127 |  | 111.0 [81.0, 157.0] | 112.0 [81.0, 157.0] | 0.033 |
| **Bicarbonate, mmol/L** | 24.2 [21.1, 27.9] | 23.9 [20.9, 27.6] | 0.041 |  | 24.2 [21.1, 27.9] | 23.9 [20.9, 27.6] | 0.041 |
| **UPCR, g/g** | 0.2 [0.1, 0.6] | 0.1 [0.0, 0.5] | 0.007 |  | 0.2 [0.1, 0.6] | 0.1 [0.0, 0.5] | 0.007 |
| **RASi** | 2076 (76.6) | 6999 (50.9) | 0.554 |  | 63.8 | 55.4 | 0.171 |
| **Beta blockers** | 1828 (67.4) | 5137 (37.4) | 0.631 |  | 50.6 | 42.7 | 0.159 |
| **CCBs** | 1996 (73.6) | 7909 (57.5) | 0.344 |  | 73.9 | 60.7 | 0.285 |
| **SGLT2is** | 242 (8.9) | 615 (4.5) | 0.179 |  | 5.4 | 5.1 | 0.013 |
| **GLP1RAs** | 35 (1.3) | 102 (0.7) | 0.055 |  | 1.3 | 0.7 | 0.055 |
| **Diuretics** | 2137 (78.8) | 3433 (25.0) | 1.279 |  | 37.7 | 34.1 | 0.077 |
| Data are presented as n (%) or median and interquartile range before IPTW.  Data are presented as % or median and interquartile range after IPTW.  *Abbreviation*s: BUN, blood urea nitrogen; Ca, calcium; CAD, coronary artery disease; CCBs, calcium channel blockers; CVA, cerebrovascular accident; eGFR, estimated glomerular filtration rate; GLP1RAs, glucagon-like peptide-1 receptor agonists; HbA1c, hemoglobin A1c; HDL, high-density lipoprotein; Hgb, hemoglobin; IPTW, inverse probability of treatment weighting; K, potassium; LDL, low-density lipoprotein; Na, sodium; PAD, peripheral artery disease; RASi, renin-angiotensin system inhibitors; SGLT2is, sodium-glucose cotransporter-2 inhibitors; SMD, standardized mean difference; TG, triglycerides; UPCR, urine protein-to-creatinine ratio. | | | | | | | |

**Supplementary Table S10. Risks of end-stage renal disease, major adverse cardiovascular events, all-cause mortality, and severe hyperkalemia between spironolactone users and nonusers in the IPTW-adjusted cohort**

| **Outcomes** | **HR (95% CI)** | **P** | **Adjusted HR**^a^ **(95% CI)** | **P** |
| --- | --- | --- | --- | --- |
| **End-stage renal disease**^b^ | 0.94 (0.71－1.25) | 0.673 | 0.90 (0.67－1.21) | 0.485 |
| **MACEs** | 0.85 (0.74－0.99) | 0.032 | 0.85 (0.73－0.97) | 0.021 |
| **Myocardial infarction** | 1.70 (1.32－2.21) | <0.001 | 1.64 (1.24－2.17) | <0.001 |
| **Stroke** | 0.88 (0.76－1.03) | 0.108 | 0.80 (0.69－0.94) | 0.006 |
| **Cardiovascular death** | 0.79 (0.57－1.10) | 0.165 | 0.78 (0.56－1.08) | 0.131 |
| **All-cause mortality** | 1.29 (1.12－1.49) | <0.001 | 1.36 (1.17－1.59) | <0.001 |
| **Severe hyperkalemia** | 1.75 (1.51－2.03) | <0.001 | 1.44 (1.24－1.68) | <0.001 |
| ^a^Adjusted hazard ratios were estimated using IPTW-adjusted Cox regression models, with additional adjustment for age, hypertension, RASi, beta-blockers, and calcium channel blockers.  ^b^End-stage renal disease was defined as initiation of long-term dialysis or kidney transplantation.  Abbreviations: CI, confidence interval; HR, hazard ratio; MACE, major adverse cardiovascular events; RASi, renin-angiotensin system inhibitors. | | | | |
